# Fructan synthesis, accumulation, and polymer traits. I. *Festulolium* chromosome substitution lines

**DOI:** 10.3389/fpls.2015.00486

**Published:** 2015-07-08

**Authors:** Joe A. Gallagher, Andrew J. Cairns, David Thomas, Adam Charlton, Peter Williams, Lesley B. Turner

**Affiliations:** ^1^Institute of Biological, Environmental and Rural Sciences, Aberystwyth UniversityAberystwyth, UK; ^2^Biocomposites Centre, Bangor UniversityBangor, UK; ^3^Department of Chemistry, Glyndwr UniversityWrexham, UK

**Keywords:** fescue (*Festuca*), genetic control, genetic variation, polymer chain length, ryegrass (*Lolium*), water-soluble carbohydrate

## Abstract

The fructans found as storage carbohydrates in temperate forage grasses have a physiological role in regrowth and stress tolerance. They are also important for the nutritional value of fresh and preserved livestock feeds, and are potentially useful as feedstocks for biorefining. Seasonal variation in fructan content and the capacity for *de novo* fructan synthesis have been examined in a *Festulolium* monosomic substitution line family to investigate variation in the polymers produced by grasses in the ryegrass-fescue complex. There were significant differences between ryegrass and fescue. Fescue had low polymeric fructan content and a high oligomer/polymer ratio; synthesis of polymers longer than degree of polymerization 6 (DP6) from oligomers was slow. However, extension of polymer length from DP10/DP20 upward appeared to occur relatively freely, and, unlike ryegrass, fescue had a relatively even spread of polymer chain lengths above DP20. This included the presence of some very large polymers. Additionally fescue retained high concentrations of fructan, both polymeric and oligomeric, during conditions of low source/high sink demand. There were indications that major genes involved in the control of some of these traits might be located on fescue chromosome 3 opening the possibility to develop grasses optimized for specific applications.

## Introduction

The fructan polymers found as storage carbohydrates in temperate forage grasses (Pollock and Cairns, 1991) have widely reported physiological roles in regrowth and stress tolerance (Vijn and Smeekens, 1999; Chalmers et al., 2005). Grasses from the ryegrass-fescue complex are well suited to the prevailing climatic conditions in many temperate regions (Wilkins and Humphreys, 2003) but do differ in nutritional value and stress tolerance. Fescues generally have lower nutritional value, but greater stress tolerance and will grow on more marginal land (Thomas and Humphreys, 1991; Humphreys et al., 1997). Fructans also play an important part in contributing to the nutritional value of forage. In many temperate regions of the world grasslands support livestock production, and in the UK around 75% of feed requirements for cattle and sheep are obtained from such grasses (Wilkins and Humphreys, 2003). Furthermore, in the future they have the potential to be useful as feedstocks for biorefining as there is an increasing demand to move from oil-based processes to sustainable carbohydrate-based processes (Van Ree and Annevelink, 2007).

The average chain length of polymeric fructan varies between species (Vijn and Smeekens, 1999). Perennial ryegrass fructan has been shown to consist of a mixed range of β(2,1) and β(2,6) molecules varying in chain length from 3 to ∼90 units (Gallagher et al., 2007), but there appears to be little information on variation in and regulation of the fructan polymer pool in the plant and any consequences of such variation for plant processes or applications. Different polymer pools may have different activities and properties. Fescue fructan has been reported to be of both generally larger and smaller molecular weight than ryegrass fructan, although this may depend on climatic conditions, the time of year at which samples are taken or the type of tissue sampled (Pollock and Jones, 1979; Spollen and Nelson, 1988; Shewmaker et al., 2006; Kagan et al., 2011). The WSC content of perennial ryegrass herbage from field plots varied over the growing season and was highest in spring (Humphreys, 1989). As there is often a high correlation between fructan and WSC contents (fructan content is high when WSC content is high: McGrath, 1988; Turner et al., 2006) fructan content may follow a similar pattern.

A *Festulolium* monosomic substitution line family is available (Harper et al., 2011) which provides ideal material to investigate and characterize variation in the fructan polymers produced by ryegrass and fescue. Fescue chromosomes have been introgressed into ryegrass to produce seven substitution lines, each of which has one whole fescue chromosome. For example, substitution line C1 has one fescue chromosome1, one ryegrass chromosome1 and 6 other (C2–C7) ryegrass chromosome pairs. All seven chromosomes of the species are represented. If trait expression is similar in the fescue parent and one of the substitution lines, then this provides preliminary information that some genetic control of the trait is located on this chromosome. The work described here was designed to investigate (1) seasonal variation in fructan content and fructan polymer length and (2) whether fescue germplasm shows significant variation from ryegrass in fructan accumulation and polymer traits including polymer synthesis, polymer-size range and polymer-size distribution. *De novo* fructan synthesis was included to provide some information on the role of synthetic processes in determining the polymer complement of the fructan pool, which is a product of both synthetic and catabolic processes.

## Materials and Methods

### Plant Material

The *Festulolium* substitution line family [sublines C1–C7 plus the F1 hybrid and the parental plants (Harper et al., 2011)] was used in the study. The first cross in the production of the family was between the tetraploid perennial ryegrass cultivar Meltra (Belgian late-flowering tetraploid) and meadow fescue (*Festuca pratensis*, Huds) line Bf1183. An F1 plant was backcrossed to the diploid ryegrass cultivar Liprior (German early flowering diploid) to produce the seven diploid monosomic substitution lines. The plants were maintained in a frost-free, unlit glasshouse in 13-cm diameter pots and renewed each year from a small group of tillers.

For the seasonal variation experiment, material was sampled during early afternoon from individuals of the parental plants and each line in the glasshouse over the 2011 growing season. Maximum and minimum daily temperature from the on-site meteorological station is shown on Supplementary Figure S1. The glasshouse was well ventilated every day to avoid high temperatures, and closed at night if low temperatures occurred. The harvest dates were: April 19, June 6, July 5, August 2, September 20, and November 1. Plant position was changed after every harvest except the first. Reproductive state was noted on each occasion and any flowering heads removed and discarded. Top growth was then removed back to a stubble height of 4 cm. This material was immediately frozen in liquid nitrogen, stored at –80°C, freeze-dried, and then finely chopped prior to extraction for WSC. As the whole plant was cut back during sampling, the material taken at subsequent harvest dates consisted of totally new growth. Leaf growth and WSC content are virtually independent of any basal carbohydrate reserves by 6 days after defoliation (Morvan-Bertrand et al., 1999; Turner et al., 2001) so each date can be treated as an independent measurement.

*De novo* fructan synthesis from sucrose was examined in triplicate with the excised-leaf induction system (Cairns and Pollock, 1988; Pollock and Cairns, 1991; Cairns et al., 2002). Uninduced leaves of the same developmental stage can be taken at any time of year for analysis in controlled environments with this system and give comparable results. Leaves are characterized as uninduced by the absence of sucrose, at which stage the enzymes of fructan synthesis are not present All the parental plants and sub-lines were shaded to <20% ambient irradiance for 7 days in the glasshouse. All fully expanded green leaves were removed and stood in water in a closed box. Time zero samples were taken, wiped dry and immediately frozen in liquid nitrogen. Leaves for induction were transferred to 25 ml conical flasks containing 20 ml 200 mM sucrose. The flasks were arranged randomly within the replicate blocks in a growth cabinet at 20°C and 400 μmol/m^2^/s irradiance for 24 h. Twenty-four-hour samples were removed from the flasks, washed thoroughly in clean water, wiped dry and immediately frozen in liquid nitrogen. All samples were stored at –80°C, freeze-dried, and then finely chopped prior to extraction for WSC.

### Carbohydrate Analysis

Extraction and analysis of the individual components of WSC for quantification was modified from Turner et al. (2002). To allow for the more heterogeneous nature of the samples, triplicate extracts were prepared in water containing 40 mg/ml sorbitol internal standard. These were heated at 100°C for 5 mins, filtered, rapidly deionized with both anion and cation exchange resins (strongly basic anion exchanger Dowex 2x8-200 and strongly acidic cation exchanger Dowex 50Wx8-200; both from Sigma) and filtered again prior to HPLC analysis. The sugars were separated and quantified by isocratic HPLC on an instrument comprising an ASI-100 automated sample injector (Dionex, Camberley, UK), P580 pump (Dionex, Camberley, UK) and Chromeleon instrument interface (Dionex, Camberley, UK), on a 300 mm × 7.8 mm column of Aminex HPX87-C (BioRad, Hemel Hempstead, UK) at 85°C, protected by an inline 0.5 μm filter and a Carbo-C guard column (BioRad, Hemel Hempstead, UK). The mobile phase was degassed water at a flow rate of 0.6 ml/min. This column completely resolved fructan molecules with a DP (degree of polymerization) of three from other fructan polymers. Based on the analysis of authentic oligomer standards, short-chain fructan molecules with a DP of 4–6 have been distinguished as shoulders or pronounced tails on the main fructan peak and integrated separately from long chain polymers. In this communication DP 3, DP 4, and DP 5–6 have been described as oligofructans and DP > 6 as polymeric fructan. Sugars were detected by refractive index (Shodex RI-71 refractive index monitor) and quantified against authentic standards. All fructans were quantified against chicory inulin (Sigma). The variation between replicate extracts was very small and the data considered in this publication are leaf tissue means for the lines.

The relative distribution of polymers of different chain lengths was assessed by HPAEC. One extract from combined replicates was extracted in water at 100°C for 5 mins and filtered. Aliquots of 25 μl were injected onto a Carbopac PA100 (Dionex: 4x 250 mm) column at 30°C and eluted with 1.0 ml/min 100 mM sodium hydroxide and a discontinuous linear gradient from 0 to 1.0 M sodium acetate. Sugars were detected by pulsed amperometry using an ED40 electrochemical detector. The relationship of DP against retention time for the analytical system used in this study was characterized with authentic inulin (β2,1-linked) oligo-and poly-saccharide standards in the range of DP3 to DP75 (Supplementary Figure S2). This method provided qualitative rather than quantitative data as the detector response is different for each individual carbohydrate species and detector response decreases with increased chain length (Cairns, 2003). Nevertheless, the pattern of peak distribution for different degrees of polymerization can be compared between lines and some parameters extracted for further analysis. Peak height was used for these comparisons as it was easy to determine. Because of detector sensitivity changes some relationships characterized in this way for different size polymers will not be accurate in absolute terms, but any differences identified between lines will be valid. Peak height, standardized against an internal control of DP10 peak height and plotted against DP gave a linear relationship (fitted regression lines were significant at a level of at least *P* < 0.05) and this relationship was used to characterize a polymer-size profile for each line. This analysis provides a novel technique to investigate fructan polymer pools and accumulation traits.

### Statistical Analysis

Analyses of variance were carried out with the standard menu-driven procedures included in GenStat^®^ for Windows^®^, Version 13.2 (Payne et al., 2010) as described in the results. Post-ANOVA multiple comparison tests were performed with Tukey. Correlations were calculated as the product moment correlation coefficient for pair-wise combinations. The MLP 3.08 (Ross, 1987) was used for curve fitting and to carry out parallel curve analysis. Pair-wise multiple comparisons are not possible with this software but selected individual pair-wise test were carried out with subsets of the data.

## Results

### Seasonal Variation in Fructan Content

**FIGURE 1:**
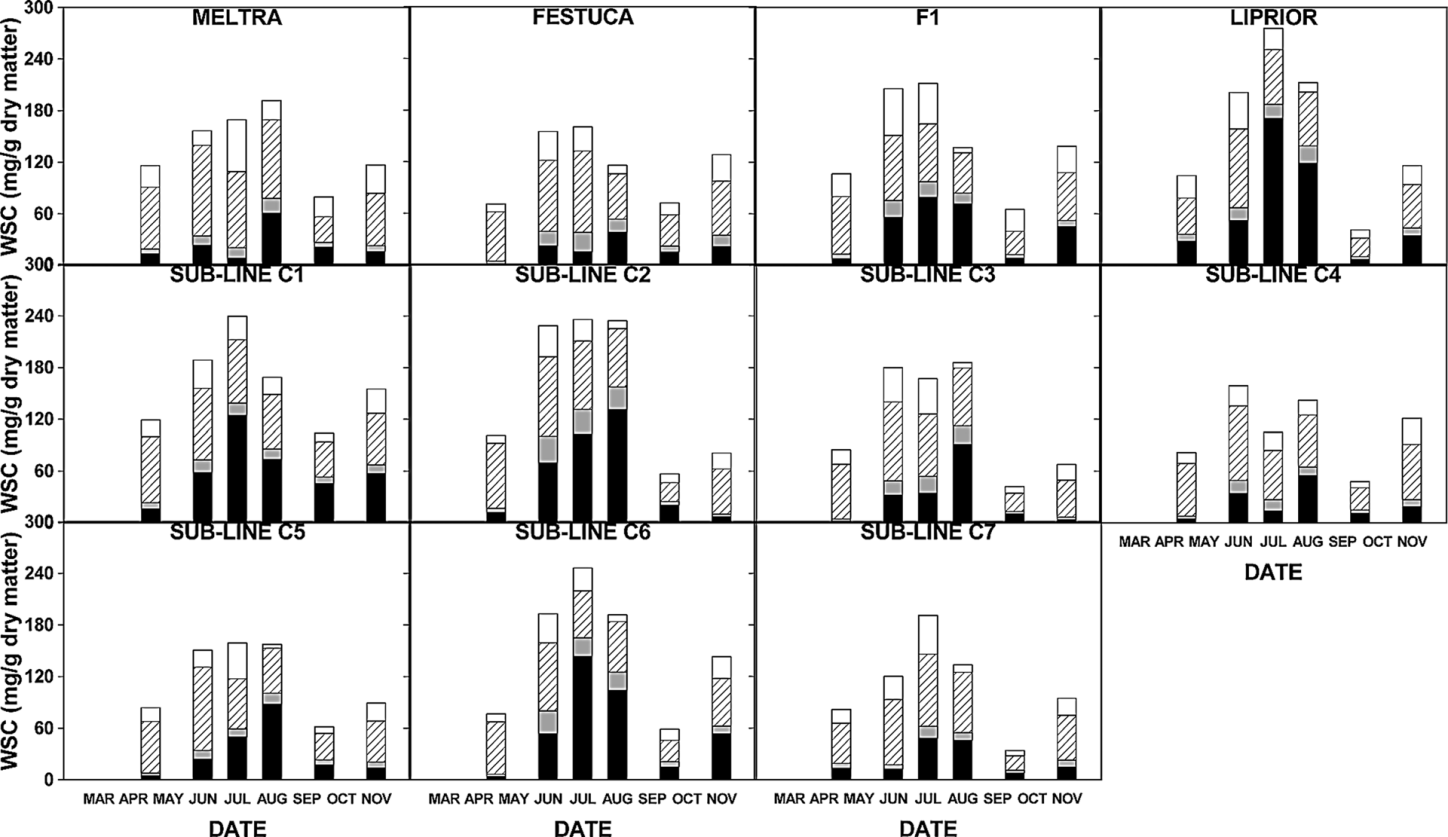
**Carbohydrate contents (mg/g DM) of leaf material from the chromosome substitution lines and their parents over the growing season from mid-April to October.** X axis labels are March, April, May, June, July, August, September and October. Polymeric fructan, black solid blocks; oligomeric fructan, gray solid blocks; disaccharides, hatched blocks; monosaccharides, open blocks. Total WSC, whole bars. Data are individual values for each line.

**FIGURE 2:**
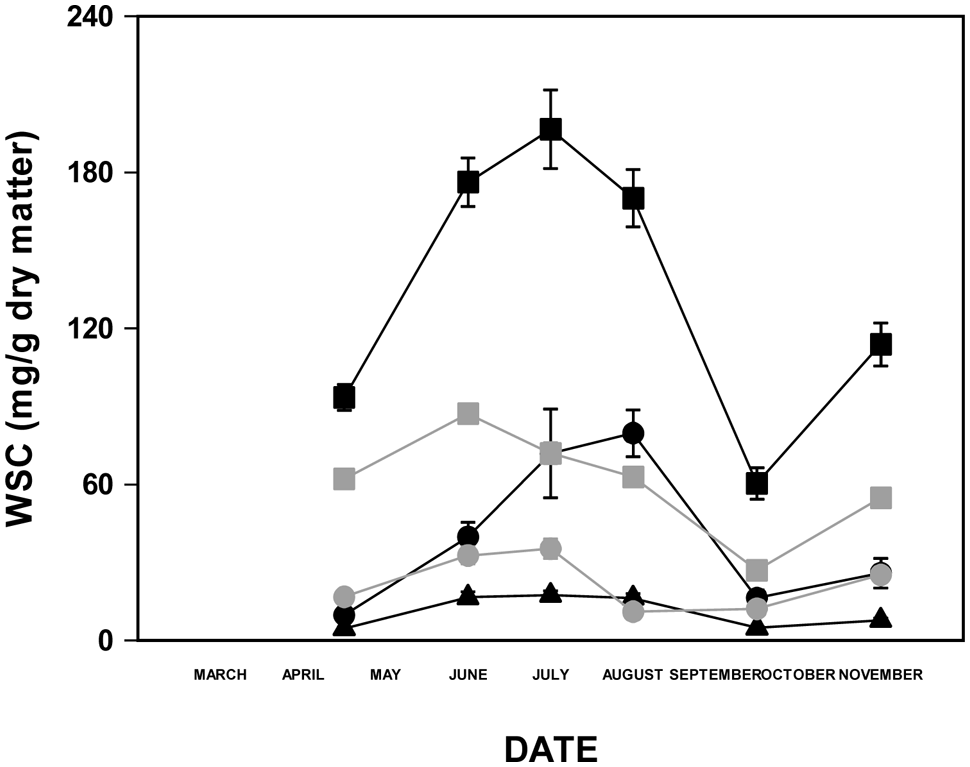
**Carbohydrate contents (mg/g DM) of leaf material from the chromosome substitution lines and their parents over the growing season.** Total WSC, black closed squares; polymeric fructan, black closed circles; oligomeric fructan, black closed triangles; disaccharides, gray closed squares; monosaccharides, gray closed circles. Data are means and SE; *n* = 11.

The vegetative material removed above a 4 cm cut was predominantly leaf blade with a small percentage of leaf sheath present. The mean total WSC content of this material varied over the growing season in all lines (**Figure 1**). The significance of these seasonal effects was tested by fitting curves to the data. The curves of best fit (those where the majority of the variation was explained by the curve equation parameters and with the lowest error mean square values) for mean sugar contents were all non-linear, confirming significant variation over the growing season. Third order polynomial curves were the best fit for disaccharide and monosaccharide sugars, but fourth order equations improved the fit for total WSC, polymeric fructan and oligomeric fructan (**Table 1**). There were no significant differences between the different substitution lines in curve form (as tested with parallel curve analysis using the polynomial curve of best fit for each sugar), indicating that the seasonal pattern was consistent. WSC was highest in the summer during June, July and August (**Figure 2**). Sucrose constituted the majority of the non-fructan sugar pool and was highest in June when it was the major sugar. Polymeric fructan content peaked later, in August, and was the major sugar in leaf material at this time. The significance of variation over the growing season was further assessed by one-way ANOVA treating date as the fixed effect and without blocking. The effect of date was significant at *P* < 0.001 for all sugars. The data for total WSC and fructan fell into two distinct groups: one group comprising June, July, and August, with high carbohydrate content, and a second group for April, September, and November, with lower carbohydrate content. With the exceptions of polymeric fructan in June and oligomeric fructan in September, carbohydrate contents were not significantly different from each other within these groups, but were all different from those of the other group.

**Table 1:** Curve fitting and analysis for leaf sugar contents over the growing season.

		Carbohydrate				
		**Total WSC**	**Polymeric fructan**	**Oligomeric fructan**	**Disaccharide**	**Monosaccharide**

**Polynomial curve form**		**Error mean square**				
Second order		2930.8	570.9	27.4	434.2	174.3
Third order		854.9	638.7	5.9	51.4	55.8
Fourth order		21.6	23.7	2.8	66.0	100.2
		
**Curve equation**		**Polynomial**				
		**Fourth order**	**Fourth order**	**Fourth order**	**Third order**	**Third order**
		
Displacement	F	3.405	2.974	4.307	1.490	0.839
	*P*	*P* < 0.05	*P* < 0.05	*P* < 0.05	NS	NS
Curve form	F	0.952	0.711	1.843	0.499	0.193
	*P*	NS	NS	NS	NS	NS

*Error mean square values for fitting different polynomial curves to mean carbohydrate content. n = 11. F-values for parallel curve analysis, fitting the curve form indicated to data for individual lines*.

There were significant effects of displacement (*P* < 0.05) for total WSC and fructan, both oligomeric and polymeric (parallel curve analysis: **Table 1**), indicating significant differences in the content of these sugars between lines. Therefore analysis of differences in sugar content between lines was investigated further by one-way ANOVA treating genotype as the fixed effect and date as a random effect (**Table 2**). The fescue parent had a low polymeric fructan content, contributing to observed lower total fructan and total WSC contents. A higher oligofructan content, led to a high oligomeric to polymeric fructan ratio. Chromosome substitution lines C4, C5, and C7 also had low sugar contents while lines C1, C2, and C6 had high sugar content. Chromosome substitution line 3 had low polymeric fructan, but moderate oligomeric fructan contents leading to a relatively high oligomeric to polymeric fructan ratio as in the fescue parent.

**Table 2:** Mean leaf sugar contents (mg/g DM) of *Festulolium* chromosome substitution lines over the growing season.

Line	Total WSC	Total fructan	Oligomeric fructan	Polymeric fructan	Ratio oligomeric/polymeric
Meltra	138.4 ab	33.6 a	9.7 ab	24.0 ab	0.546 ab
Fescue	117.1 ab	31.9 a	13.2 ab	18.7 a	1.024 b
F1	144.0 ab	56.1 a	11.3 ab	44.8 ab	0.347 a
Liprior	158.6 ab	80.7 a	12.0 ab	68.7 b	0.261 a
Subline C1	162.6 b	73.7 a	11.2 ab	62.4 ab	0.227 a
Subline C2	156.4 ab	73.6 a	16.5 b	57.1 ab	0.338 a
Subline C3	121.3 ab	39.9 a	11.1 ab	28.8 ab	0.655 ab
Subline C4	109.6 a	31.7 a	8.8 ab	22.9 ab	0.492 ab
Subline C5	117.0 ab	41.1 a	8.2 a	32.9 ab	0.381 a
Subline C6	151.6 ab	76.8 a	14.5 ab	62.2 ab	0.388 a
Subline C7	109.2 a	31.2 a	7.5 a	23.7 ab	0.385 a
Probability	*P* = 0.002	*P* < 0.001	*P* = 0.012	*P* < 0.001	*P* = 0.002
LSD (0.05)	31.47	29.80	4.79	27.53	0.3439

*Probability and estimates of LSD for the means at the 5% level from one-way ANOVA treating plant as a fixed effect. n = 6. Means in columns followed by the same letter are not significantly different at the 5% level according to post hoc multiple pair-wise Tukey tests*.

### Fructan Synthesis

**FIGURE 3:**
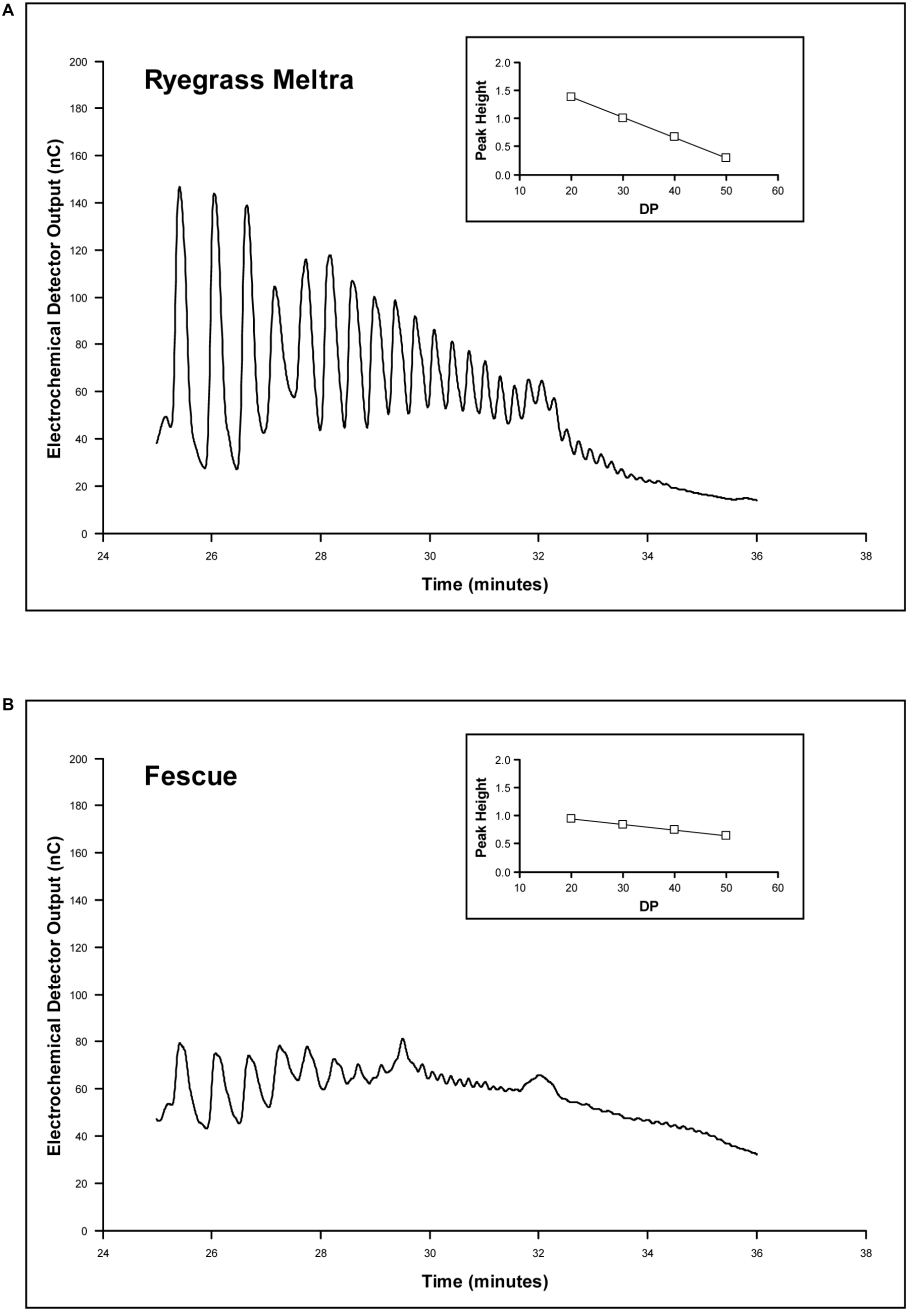
**HPAEC chromatograms for (A) the Meltra ryegrass parent and (B) the fescue parent plant for retention times between 25 and 35 min.** Insets show the regression of peak height on polymer chain length for DP20 to DP50.

The capacity to synthesize fructan *de novo* from sucrose was tested with the excised leaf induction system. Leaf carbohydrate content was initially depleted by 7 days at low light and there was considerable variation between lines in the capacity to retain fructan under conditions where photosynthesis was considerably reduced (**Table 3**). Fructan retention was significantly higher (*P* < 0.05) in fescue and chromosome substitution line C3, and lower in most other lines. Liprior retained a high proportion of total fructan as oligofructan. There was little or no sucrose or monosaccharide present and the leaves were considered uninduced. Fructan synthesis was performed with exogenously supplied sucrose in the light to ensure no limitation of sucrose supply even if plants had low photosynthetic capacity as well as full expression of all light-induced genes. As the quantity of fructan synthesized never exceeded 35% of combined assimilation and uptake of sucrose (leaf sucrose content at *t* = 24 h), it is unlikely that any limitation of carbon supply occurred during the experiment. Fescue showed low fructan synthetic capacity, and particularly low conversion of oligomer into polymer (**Table 4**), but this low conversion rate was not seen in any of the chromosome substitution lines. Chromosome substitution lines C1 and C6 had lower synthetic rates than lines C3 and C7. Information on the relative distribution of different chain lengths in the high molecular weight fructan fraction was assessed from the pattern of polymer peaks in the range from DP 10 upward on the HPAEC chromatograms. The major polymer in this range was DP 20 for all lines (**Table 4**). There were obvious differences in the distribution of polymers in the range DP 20–DP 50. The derived relationship of peak height to DP (calculated from retention time; Supplementary Figure S2) was used to characterize a polymer-size profile for each line. There was variation for both profile regression constant and profile slope as illustrated by the contrasting data for the Meltra ryegrass parent and the fescue parent shown on **Figure 3**. Parallel curve analysis with the MLP 3.08 confirmed there were significant differences (*P* < 0.001) between lines for both parameters (**Table 4**). Based on pair-wise tests, fescue had a smaller profile regression slope and constant than ryegrass (*P* < 0.05). The F1 hybrid was intermediate with a significantly different profile constant (*P* < 0.05) from fescue and both ryegrass parents and a significantly different slope (*P* < 0.05) from fescue and Meltra. Chromosome substitution lines C1 and C6 had high regression constants similar to the ryegrass parents and significantly different (*P* < 0.05) from both lines C2 and C3. The regression slope of chromosome substitution lines C2 and C3 was significantly lower (*P* < 0.05) than line C1, indicating a relatively high proportion of very large polymers as in the fescue parent. Although there may have been variation in the largest polymer formed during the 24 h synthetic period it was not possible to test this from the data (**Table 4**).

**Table 3:** Mean leaf fructan contents (mg/g DM) of *Festulolium* chromosome substitution lines after depletion of carbohydrates at low light.

Line	Total fructan	Oligomeric fructan	Polymeric fructan	Ratio oligomeric/polymeric
Meltra	0.7 a	0.0 a	0.7 a	0.000 a
Fescue	33.1 c	9.4 c	23.7 c	0.391 ab
F1	2.2 a	0.6 a	1.6 a	0.273 ab
Liprior	4.8 a	2.0 a	2.9 a	0.660 b
Subline C1	0.5 a	0.0 a	0.5 a	0.000 a
Subline C2	5.6 a	1.9 a	3.8 a	0.498 ab
Subline C3	20.1 bc	5.1 bc	15.0 bc	0.338 ab
Subline C4	9.2 ab	1.6 ab	7.5 ab	0.191 ab
Subline C5	1.0 a	0.0 a	1.0 a	0.000 a
Subline C6	1.7 a	0.2 a	1.5 a	0.102 ab
Subline C7	3.5 a	0.6 a	2.9 a	0.161 ab
Probability	*P* < 0.001	*P* < 0.001	*P* < 0.001	*P* = 0.008
LSD (0.05)	8.07	2.72	5.51	0.3438

*Probability and LSD estimates from one-way ANOVA treating plant as the fixed effect and replicate as a random effect. n = 3. Means in columns followed by the same letter are not significantly different at the 5% level according to post hoc multiple pair-wise Tukey tests*.

**Table 4:** *De novo* fructan synthesis in the *Festulolium* chromosome substitution lines.

Line	Fructan synthesis				Major polymer	Polymer profile between DP20 and DP50 Parameters for regression of peak height on chain length		Largest polymer
		

	Total fructan	oligomers	polymers	oligomer/polymer ratio	>DP10		

	**mg/g DM/24 h**					**Regression constant**	**Regression slope**	
Meltra	30.2 ab	16.8 ab	13.3 abc	1.26 a	20	2.100 ± 0.0249	–0.0362 ± 0.00068	73
Fescue	20.9 a	18.7 abc	5.2 a	3.84 b	20	1.157 ± 0.0000	–0.0105 ± 0.00000	140
F1	57.2 cd	33.8 d	23.4 cd	1.45 a	20	1.504 ± 0.1050	–0.0274 ± 0.00285	65
Liprior	19.5 a	10.4 a	9.1 ab	1.24 a	20	2.364 ± 0.2590	–0.0364 ± 0.00706	90
Subline C1	33.8 abc	19.2 abc	14.6 abc	1.32 a	20	2.325 ± 0.0805	–0.0325 ± 0.00219	80
Subline C2	49.5 bcd	30.5 cd	19.0 bcd	1.60 a	20	1.241 ± 0.1440	–0.0198 ± 0.00393	100
Subline C3	62.7 d	32.3 d	30.4 d	1.10 a	20	1.477 ± 0.0650	–0.0204 ± 0.00177	100
Subline C4	44.7 abcd	25.0 bcd	19.7 bcd	1.31 a	20	1.925 ± 0.0712	–0.0275 ± 0.00194	90
Subline C5	38.2 abcd	19.0 abc	19.2 bcd	0.97 a	20	1.959 ± 0.1270	–0.0247 ± 0.00346	80
Subline C6	33.9 abc	16.3 ab	17.7 abcd	0.96 a	20	2.475 ± 0.2000	–0.0308 ± 0.00545	100
Subline C7	54.4 bcd	33.1 d	21.2 bcd	1.56 a	20	1.473 ± 0.0235	–0.0235 ± 0.00276	90
Mean	40	23	18	1.5	20	1.818 ± 0.1414	–0.0263 ± 0.00230	92
Probability	*P* < 0.001	*P* < 0.001	*P* < 0.001	*P* < 0.001		*P* < 0.001	*P* < 0.001	
LSD (0.05)	14.91	6.95	7.79	1.02				

*Mean leaf fructan synthesis (mg/g DM) of detached leaves during 24 h in the presence of exogenously supplied sucrose in the light. Probability and LSD estimates from one-way ANOVA treating plant as the fixed effect and replicate as a random effect. n = 3. Means in columns followed by the same letter are not significantly different at the 5% level according to post hoc multiple pair-wise Tukey tests. Polymer traits (the major polymer > DP10, polymer profile between DP20 and DP50 and the largest observed polymer) are from single chromatograms after pooling the replicate extracts and regression parameters have been compared by parallel curve analysis with the MLP 3.08*.

### Polymer-Size Profile in Leaf Tissue from Growing Plants

The polymer-size profile was also examined by HPAEC chromatography for the April, July, and November samples from the seasonal experiment. One-way ANOVA, treating date as the fixed effect and without blocking, showed a significant (*P* < 0.001) effect of date on the major and largest polymers present and on the regression parameters for the relationship of peak height to DP. There were no significant differences between April and November for any polymer-size trait, and polymer size was of the same order as in the induction experiment (see **Table 4**) with a mean DP of 22 for the major peak and a mean DP of 95 for the largest polymer observed. Pair-wise tests showed that on both dates the fescue parent and chromosome substitution line C3 again had lower profile regression constants (*P* < 0.05) than line C1 and, with the exception of a non-significant difference between the fescue parent and chromosome substitution line C1 in November, lower profile regression slopes (*P* < 0.05) as well. Compared with this spring and autumn data, the samples from July had significantly higher (*P* < 0.001) mean DP values for the major and largest polymers present and for the regression parameters of the peak height against DP relationship (**Table 5**). The profile regression lines for this data were calculated for the portion of the polymer-size profile consistently past the major peak in all cases; that is for the DP range 50–90. The polymer size data for July were closely correlated to the increased fructan content at this time. Correlation coefficients for the polymer-size traits and high molecular weight fructan content were calculated using data from all three dates and were highly significant (*P* < 0.001) in all cases (major peak, *r* = 0.733; largest peak, *r* = 0.693; profile regression constant, *r* = 0.700; profile regression slope, *r* = 0.658; *n* = 33). However, the increased DP of the major and largest polymers present was not observed in all lines. The fescue parent showed no increase and even a possible decrease in the largest polymer detected. If the lines are ranked on largest polymer present for each of the datasets, then the fescue parent moves from the top line in the induction experiment and in April to the bottom in July. This also applies to chromosome substitution line C3. The fescue parent had a lower profile regression slope than chromosome substitution line C3 (*P* < 0.05) and both had lower slopes than line C1 and Liprior (*P* < 0.05). Fescue and line C3 had lower profile regression constants than line C1 and Liprior (*P* < 0.05). Although there were no significant differences between the data for the two ryegrass parents Meltra and Liprior in the induction experiment, fructan metabolism differed over course of the growing season in these lines. In pair-wise tests the regression parameters for Meltra and Liprior were significantly different (*P* < 0.05) in July. The regression slopes were not significantly different in April and November although the regression constants were different (*P* < 0.05) on both these dates, probably reflecting the annual lower (*P* < 0.05) high molecular weight, polymeric fructan content of Meltra (**Table 2**).

**Table 5:** Polymer traits for July samples.

Line	Major polymer	Polymer profile between DP50 and DP90		Largest polymer

	>DP10	Parameters for regression of peak height on chain length		
		Regression constant	Regression slope	
Meltra	23	0.529 ± 0.0752	–0.0029 ± 0.0011	96
Fescue	23	1.540 ± 0.1650	–0.0140 ± 0.0023	90
F1	45	5.878 ± 0.3650	–0.0605 ± 0.0051	127
Liprior	48	5.784 ± 0.3450	–0.0550 ± 0.0048	140
Subline C1	56	6.208 ± 0.6460	–0.0496 ± 0.0091	140
Subline C2	42	5.307 ± 0.3620	–0.0556 ± 0.0051	140
Subline C3	22	2.482 ± 0.2250	–0.0275 ± 0.0032	90
Subline C4	48	7.551 ± 0.4080	–0.0762 ± 0.0057	127
Subline C5	56	7.038 ± 0.8790	–0.0615 ± 0.0123	150
Subline C6	56	5.200 ± 0.4720	–0.0378 ± 0.0066	140
Subline C7	42	3.213 ± 0.2470	–0.0332 ± 0.0035	140
Mean	42	4.612 ± 0.6972	–0.0431 ± 0.0067	125
Probability		*P* < 0.001	*P* < 0.001	

*The major polymer >DP10, polymer profile between DP50 and DP90 and the largest observed polymer are from single chromatograms. The regression parameters have been compared by parallel curve analysis with the MLP 3.08*.

## Discussion

### Fructan Synthesis and Seasonal Variation

During *de novo* synthesis of fructan from sucrose, fructan oligomers initially accumulated to high levels. Guerrand et al. (1996) also reported that concentrations of low DP molecules were greater than those of high DP molecules; in their case for up to 72 h after initiation of fructan induction. In the current study polymers larger than DP6 were beginning to predominate after 24 h in most lines. The faster time-course is probably explained by the inclusion of exogenous supplied sucrose in addition to light as an inducer of fructan synthesis.

Water-soluble carbohydrate and fructan content of vegetative material above 4 cm stubble cut varied significantly over the growing season. Fructan content was highest when WSC was high but lagged behind peak WSC content. Polymer chain length was longest when fructan content was high. This is not surprising as the formation of large polymers will minimize osmotic effects of storing large amounts of carbon, and is in agreement with some other studies on temperate forage grasses. Shewmaker et al. (2006) and Jensen et al. (2014) also found fructan content was highest in summer, although McGrath (1988) and Kagan et al. (2011) reported highest concentrations in spring. Fructan accumulates at low temperatures (Hisano et al., 2008) and is catabolized at warm temperatures in low light and short days (Cairns et al., 2002), so differences in fructan content at the beginning of the growing season could result from the degree of cold-exposure experienced over the previous winter. In addition, flowering is well-known to affect sugar metabolism in grasses (Pollock and Cairns, 1991) and could influence the timing of peak WSC content. In the current work no reproductive material was included in the samples. The Meltra ryegrass parent, the fescue parent, the F1 and chromosome substitution line C3 did not flower during the sampling period. Peak flowering was June to early July for other plants (data not shown). Since no significant differences in the seasonal pattern of change in sugar content between lines were found, it would seem unlikely that flowering time has confounded the WSC data of this study.

The methods used in this study have provided data suitable for our purposes of comparing lines. Recently Harrison et al. (2009, 2011) have described a method which gives improved quantification and identification of individual isomers. However, this is currently limited to fructans up to DP100, so although its use might have provided further information on isomer variation within a DP size in this study it would not be suitable for analyzing fescue. The complement of fructan molecules present was changed by long term accumulation of fructan as the constitutive patterns of the polymer-size profiles in vegetative material above a 4 cm cut were different from those observed during fructan induction in leaf blades. This was particularly obvious in July when fructan content was high; the profiles at this time were not simply amplifications of the patterns observed in the induction experiment. It is possible that this is a consequence of a role for fructan exohydrolase in addition to fructosyltransferases in fructan synthesis as proposed by Lothier et al. (2007). The involvement of fructan breakdown and some degree of fructan turnover may lead to greater diversity in molecular structure and consequent variation in polymer-size profile. The capacity to retain fructan during periods when sink demand exceeds carbon supply could therefore also play a role.

### Genetic Variation and Control

In this germplasm opportunities for identifying possible genetic control arise when a distinct difference between the fescue and ryegrass parents can also be seen in one or more of the monosomic chromosome substitution lines, although it should be borne in mind that differences between the chromosome substitution lines could arise from segregation of the ryegrass background. Ryegrass does contain variation for relevant traits and divergence in fructan-size profiles between Meltra and Liprior was observed here during the latter part of the growing season. High sugar ryegrasses have been shown to maintain high WSC throughout the growing season (Smith et al., 1998) and to contain high fructan concentrations across a range of polymer chain lengths (Harrison et al., 2011).

There were differences between fescue and ryegrass for a number of traits: oligofructan, polymeric fructan, total fructan, total WSC, synthesis of polymers >DP6, and a high oligomeric to polymeric fructan ratio. Additionally, extension of polymer length from DP10/DP20 upward appeared to occur relatively freely. Unlike ryegrass, fescue had a relatively even spread of polymer chain lengths above DP20 including the presence of some very large polymers. Some authors have found little detectable long-chain fructan in tall fescue (Kagan et al., 2011) but Spollen and Nelson (1988) also reported low concentrations of fructan polymers across a wide range of chain lengths. There was little variation within the chromosome substitution lines for the rate of *de novo* fructan synthesis or the conversion of oligomers into polymers, which may indicate these traits are under multi-gene control spread across several chromosomes. However, many of the other ‘fescue’ traits do appear to be present in chromosome substitution line C3. The role of genes on fescue chromosome 3 in determining trait expression is supported by the mapping of two genes involved in fructan metabolism to chromosome 3 (Lasseur et al., 2006; Lothier et al., 2007; Hisano et al., 2008). 6G-FFT catalyzes the transfer of fructose from a fructan molecule to glucose (C6) of either a sucrose or a second fructan molecule forming fructans of the inulin neoseries type. 1-FEH hydrolyses β-(2,1) linkages within fructan molecules but has previously been associated with high fructan-synthetic activity (Lothier et al., 2007). Analysis of the properties of the *Festuca* forms of these enzymes might elucidate further their role in determining the fescue specific aspects of fructan metabolism described here.

### Functional Implications

Fructan pools in fescues and ryegrasses have been shown to display significant differences in polymer form. These may have functional consequences both for physiological activity within the plant and for the properties of the carbohydrate during various *in vitro* applications. It appears probable that major control of some, although not all, of the fructan metabolism traits characterized in this study resides on chromosome 3. This control could be located more precisely by analysis of the chromosome 3 substitution line recombination series. However, it is interesting that introgression of *Festuca* segments into *Lolium* chromosome 3 has also been implicated in tolerance to drought (Humphreys et al., 2005) lending support to the functional role of fructan in plants during abiotic stresses including drought (Vijn and Smeekens, 1999; Chalmers et al., 2005; Livingston et al., 2009). Accumulation of high-DP fructan has been shown to be associated with drought tolerance in cocksfoot, although there may be interactions with rooting depth (Volaire et al., 1998). Such a role would appear to exclude osmotic effects as the most important as the large fructan molecules found in fescue fructan will have lower osmotic activity. Perhaps larger polymers offer good interactions with membranes while minimizing osmotic changes. Inulins have been shown to stabilize liposome membranes during low temperature and desiccation treatments (Hincha et al., 2000). Moreover increasing fructo-oligosaccharide chain length improved membrane stability against leakage after air-drying (Hincha et al., 2002). The nutritional value of different fructan polymers does not appear to have been investigated, but fructans with different ranges of polymer chain length will be particularly suitable for different biorefining applications.

Fescue retained high concentrations of fructan during conditions of low source/high sink demand. One can speculate that this trait might make the fructan content of fescue more stable to environmental fluctuations which cause changes in source/sink demand. Predictable, stable biomass yields and/or composition are major factors for the economics of grassland use, whether as feed for livestock or for biorefining. Similar and/or different variation for fructan polymer traits may be available in diverse ryegrass germplasm. Ryegrasses exhibit better agronomic traits if good agricultural land is available, while fescues generally have lower nutritional value, but greater stress tolerance and will grow on more marginal land (Thomas and Humphreys, 1991; Humphreys et al., 1997). Alternatively, introgression of the relevant fescue chromosome 3 segment into high sugar ryegrass might produce plants containing high concentrations of fructan with an even spread of chain lengths. This work adds to the possibility of producing ‘designer’ grasses, based on varying polymeric sugar structure and content, to maximize options for multi-functional grasslands over the course of the year for different animal feed, biorefining, and land management applications.

## Author Contributions

JG, AC, and PW conceived and designed the study. AC, DT, and LT carried out the experiments and analyzed the data. JG and LT wrote the paper.

## Conflict of Interest Statement

The authors declare that the research was conducted in the absence of any commercial or financial relationships that could be construed as a potential conflict of interest.

## Abbreviations

ANOVA: analysis of variance
DM: dry matter
DP: degree of polymerization
1-FEH: fructan 1-exohydrolase
6G-FFT: fructan: fructan 6-glucose-fructosyltransferase
HPAEC: high performance anion exchange chromatography
LSD: least significant differences
MLP: maximum likelihood programme
WSC: water-soluble carbohydrate
