# Supplementary material for: Fructan synthesis, accumulation, and polymer traits. I. Festulolium chromosome substitution lines
Source: Front Plant Sci. 2015 Jul 8;6:486. doi: 10.3389/fpls.2015.00486 (PMC4495318; doi:10.3389/fpls.2015.00486)
Supplement: Supplementary file 1 [file Data_Sheet_1.PDF]

**Supporting Information**

**Figure S1**. Meteorological data. Maximum (solid line) and minimum (dashed line) daily temperature ($^oC$) over the growing season from the Institute on-site meteorological station.

**Figure S2.** Size calibration of Dionex HPAEC-PAD using inulin (β2,1-linked) oligo-and poly-saccharides in the range of DP3 to DP75 with the fitted 4th order polynomial $y = 0.00016x^4 - 0.00975x^3 + 0.24805x^2 - 2.28658x + 9.99361$; $r^2 = 0.9998$.

**Figure S1**

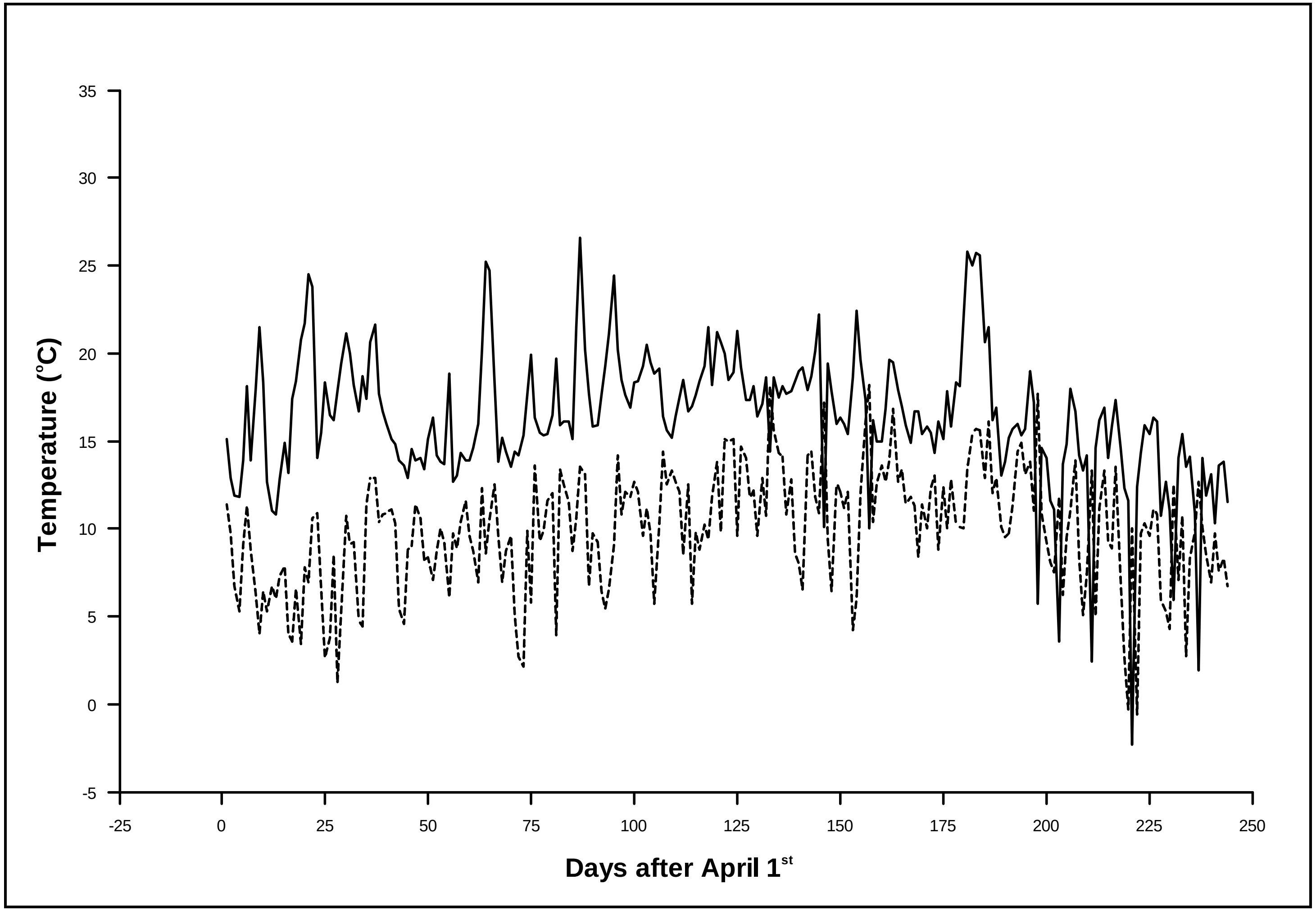

Temperature (°C)
35
30
25
20
15
10
5
0
-5
-25
0
25
50
75
100
125
150
175
200
225
250
Days after April 1st

**Figure S2**

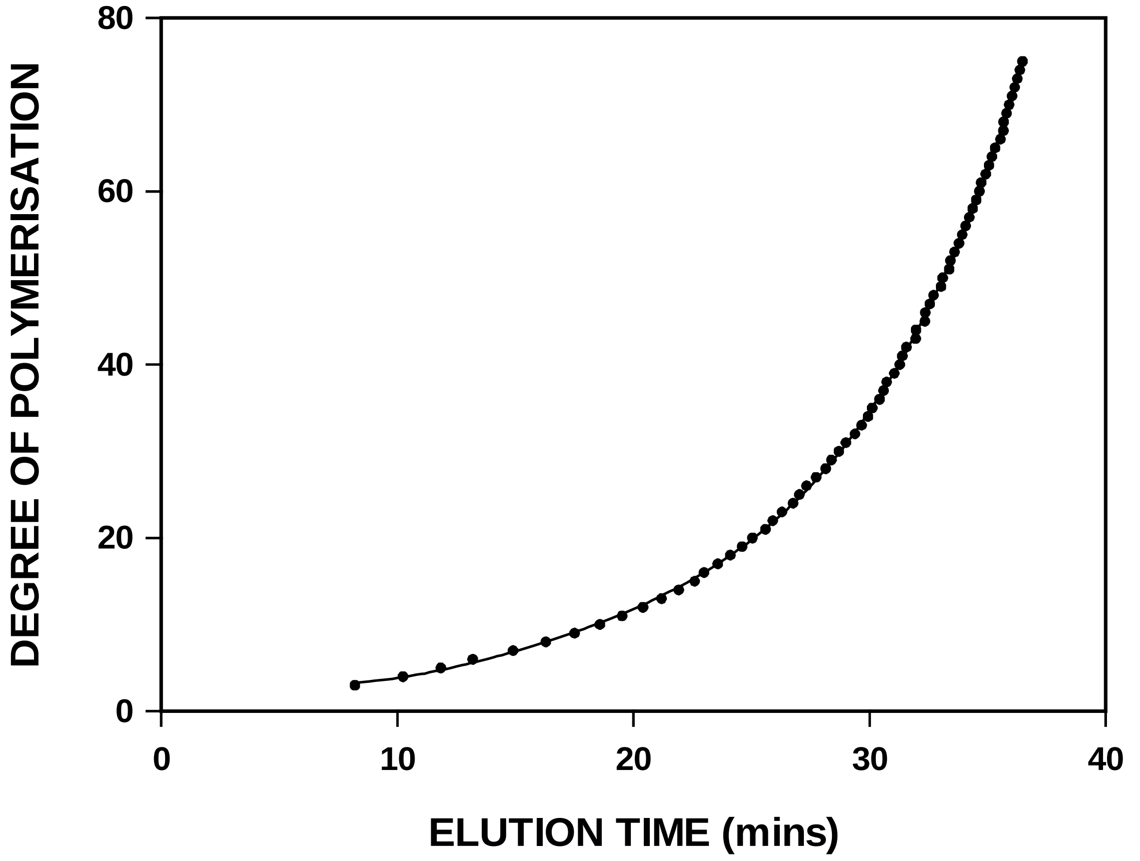

80
60
40
20
0
DEGREE OF POLYMERISATION
0
10
20
30
40
ELUTION TIME (mins)
